# The Histone Deacetylase Inhibitor Suberoylanilide Hydroxamic Acid (SAHA) Restores Cardiomyocyte Contractility in a Rat Model of Early Diabetes

**DOI:** 10.3390/ijms20081873

**Published:** 2019-04-16

**Authors:** Leonardo Bocchi, Benedetta M. Motta, Monia Savi, Rocchina Vilella, Viviana Meraviglia, Federica Rizzi, Serena Galati, Annamaria Buschini, Mirca Lazzaretti, Peter P. Pramstaller, Alessandra Rossini, Donatella Stilli

**Affiliations:** 1 Department of Chemistry, Life Sciences and Environmental Sustainability, University of Parma, 43124 Parma, Italy; leonardo.bocchi@unipr.it (L.B.); monia.savi@unipr.it (M.S.); rocchina.vilella@studenti.unipr.it (R.V.); serena.galati@unipr.it (S.G.); annamaria.buschini@unipr.it (A.B.); mirca.lazzaretti@unipr.it (M.L.); 2 Institute for Biomedicine, Eurac Research, 39100 Bolzano, Italy (affiliated institute of the University of Lübeck, 23562 Lübeck, Germany); Benedetta.Motta@eurac.edu (B.M.M.); viviana.meraviglia@gmail.com (V.M.); Peter.Pramstaller@eurac.edu (P.P.P.); 3 Department of Medicine and Surgery, University of Parma, 43126 Parma, Italy; federica.rizzi@unipr.it

**Keywords:** diabetes, HDAC inhibition, cardiomyocyte mechanics, calcium transients, cell oxidative stress

## Abstract

In early diabetes, hyperglycemia and the associated metabolic dysregulation promote early changes in the functional properties of cardiomyocytes, progressively leading to the appearance of the diabetic cardiomyopathy phenotype. Recently, the interplay between histone acetyltransferases (HAT) and histone deacetylases (HDAC) has emerged as a crucial factor in the development of cardiac disorders. The present study evaluates whether HDAC inhibition can prevent the development of cardiomyocyte contractile dysfunction induced by a short period of hyperglycemia, with focus on the potential underlying mechanisms. Cell contractility and calcium dynamics were measured in unloaded ventricular myocytes isolated from the heart of control and diabetic rats. Cardiomyocytes were either untreated or exposed to the pan-HDAC inhibitor suberoylanilide hydroxamic acid (SAHA) for 90 min. Then, a fraction of each group of cells was used to evaluate the expression levels of proteins involved in the excitation–contraction coupling, and the cardiomyocyte metabolic activity, ATP content, and reactive oxygen species levels. SAHA treatment was able to counteract the initial functional derangement in cardiomyocytes by reducing cell oxidative damage. These findings suggest that early HDAC inhibition could be a promising adjuvant approach for preventing diabetes-induced cardiomyocyte oxidative damage, which triggers the pro-inflammatory signal cascade, mitochondrial damage, and ventricular dysfunction.

## 1. Introduction

Diabetes is a risk factor for the development of various cardiovascular complications, which constitute the leading causes of morbidity and mortality in both type 1 and type 2 diabetic subjects [1]. The incidence of cardiovascular diseases is common in diabetic patients and accounts for 80% of deaths, even in the presence of a tight control over metabolic state and blood glucose levels by existing therapeutic agents. Despite extensive investigation, development of effective treatments against the complications of diabetes has proven challenging [2,3].

Hyperglycemia is a central player in activating several adaptive and maladaptive responses in myocardial tissue among which cell oxidative stress and moderate tissue inflammation constitute critical early pathogenic components [4,5,6,7,8,9]. Mitochondrial injury also has a causative role in the pathophysiology of diabetic heart disease [5,10], largely contributing to the generation of reactive oxygen species (ROS) and myocardial inflammation, leading to the development and progression of cardiac abnormalities and dysfunction. Hence, there is a need to identify novel adjuvant therapeutic approaches specifically aimed at counteracting the initial hyperglycemia-induced alterations in myocardial tissue.

In this context, histone acetyltransferases (HAT) and histone deacetylases (HDAC) have recently gained great attention as important molecules involved in the regulation of a variety of cellular responses, as well as in the modulation of pathological conditions, including diabetes and heart failure [11,12,13,14]. Histone acetylation mediated by HAT results in the modification of the structure of chromatin, leading to nucleosome relaxation and transcriptional activation. It follows that the acetylation of nucleosome histone tails provides a critical mechanism for epigenetic control of gene expression. In contrast, the reverse reaction mediated by HDACs induces de-acetylation, chromatin condensation, and transcriptional repression [15]. The interplay between HATs and HDACs, which physiologically determinates the acetylation status of histone tails, recently has emerged as a crucial factor in the development of cardiac disorders with different etiologies [2]. Additionally, proteomic studies have revealed that non-histone proteins are also subjected to reversible lysine acetylation [13], further highlighting the biological significance of this post-translational modification.

In the setting of diabetes, recent evidence indicates that HDAC inhibition increases the levels of antioxidant enzymes and reduces cardiac dysfunction in experimental models of advanced type I diabetes [16] and ameliorates cardiac performance and metabolic disturbances in murine models of both type I and type II diabetes [2,17]. An additional study [18] shows that chronic in vivo treatment of diabetic mice with a HDAC3 inhibitor, besides preventing diabetes-induced ventricular dysfunction and remodeling, maintains cardio-protective effects for at least three months after the end of treatment, like a “protective memory” phenomenon. Furthermore, Wu et al. [19] recently documented that HDAC inhibition attenuates ischemia/reperfusion cardiac injury in diabetic hearts.

Previous data, although strongly suggestive that HDAC inhibitors may constitute a potential new therapeutic tool for the treatment of diabetic cardiomyopathy, mainly are concerned with the advanced stages of diabetes. On the other hand, we previously showed that alterations in the redox state of cardiac cells and the activation of pro-inflammatory signal cascades occur from the initial stages of diabetic disease [4], resulting in the first signs of impaired cardiomyocyte contractility and calcium dynamics, well before the appearance of the overt cardiomyopathy phenotype and collagen accumulation in cardiac tissue [7,20,21].

To the best of our knowledge, the cardioprotective effects of HDAC inhibition at the very early stages of diabetes and the analysis of the potential underlying mechanisms have never been explored. To specifically address this issue, we evaluated the functional and metabolic response to HDAC inhibition of cardiomyocytes (CMs) isolated from rats with streptozotocin (STZ) induced diabetes after three weeks of hyperglycemia. The small molecule suberoylanilide hydroxamic acid (SAHA), a pan-HDACs inhibitor [15], was used. Notably, SAHA (Vorinostat) use was approved by the US FDA in October 2006 for the treatment of refractory cutaneous T-cell lymphoma [22]. Recently, we reported that SAHA-induced HDAC inhibition ameliorates intracellular Ca^2+^ dynamics and, accordingly, contractile performance in normal adult rat CMs, supporting the hypothesis that the modulation of the HAT/HDAC interplay by small active molecules can positively affect the functional properties of the CMs [23].

We found here that SAHA-induced HDAC inhibition was able to prevent the occurrence of the first signs of cardiomyocyte functional derangement in the diabetic heart mainly by reducing the accumulation of intracellular ROS. These findings may open new ways for the development of therapeutic approaches based on small molecules capable of counteracting the initial functional damage induced by diabetes in myocardial tissue.

## 2. Results

### 2.1. Effect of SAHA Treatment on Cardiomyocyte Mechanics and Calcium Transients

Ex-vivo experiments were performed to evaluate the ability of SAHA exposure to ameliorate calcium dynamics and contractile properties of CMs isolated from adult rats after a short period of hyperglycemia (3 weeks) that constitutes a time point characterized by the occurrence of the first signs of dysfunction, as measured at the cellular level [4]. Diabetic CMs were either untreated (D group) or incubated with 2.5 µmol SAHA (D + SAHA group) for 90 min.

**Figure 1:**
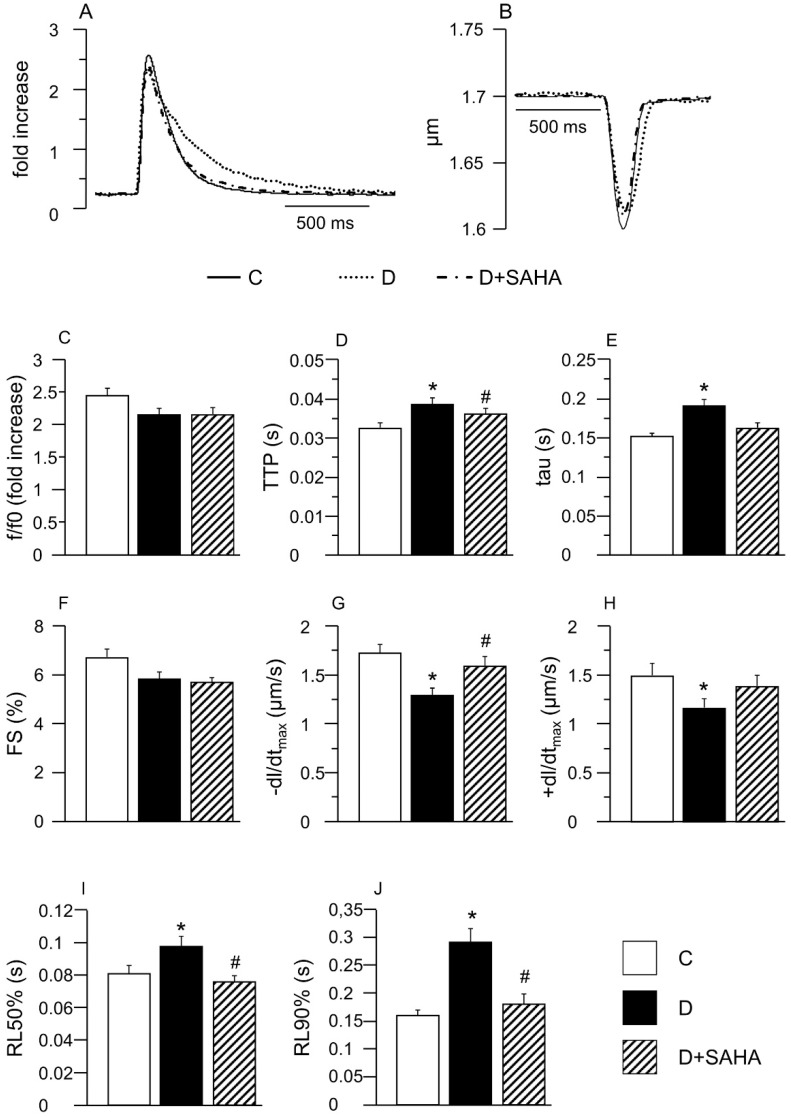
Effect of suberoylanilide hydroxamic acid (SAHA) treatment on calcium transients and cell mechanics in left ventricular myocytes. (**A**,**B**) Representative examples of calcium transients (**A**: normalized traces) and sarcomere shortening (**B**) recorded from control (C, solid line), diabetic (D, dotted line) and SAHA treated diabetic cells (D + SAHA, dashed line). In bar graphs **C**–**J**: Mean values ± SEM of the amplitude of calcium transient (**C**: f/f0), time-to-peak of the calcium transient (**D**: TTP), time constant of the rate of intracellular Ca^2+^ clearing (**E**: tau), fraction of shortening (**F**: FS), maximal rate of shortening (**G**: −dL/dt_max_), maximal rate of re-lengthening (**H**, +dL/dt_max_), time to 50% and 90% re-lengthening (**I**: RL50%; J: RL90%), measured in 45 C, 61 D, and 48 D + SAHA cardiomyocytes (CMs). * *p* < 0.05 significant differences vs. C; # *p* < 0.05 significant differences vs. D. (GLM-ANOVA for repeated measurements).

The average diastolic sarcomere length was approximately equal to 1.7 µm in both control (C group) and diabetic CMs, independent of the treatment. Conversely, in accordance with previous data [4], intracellular Ca^2+^ handling and contraction/relaxation properties measured in unloaded ventricular myocytes isolated from D hearts were partially impaired compared to C (Figure 1). Slower calcium kinetics were observed in D cells, which exhibited a prolonged time-to-peak of the calcium transient (TTP, +19%, *p* < 0.01, Figure 1D) associated with higher values of the time required for cytosolic calcium clearing (tau, +26%, *p* < 0.01, Figure 1E), in the absence of statistically significant differences in the amplitude of the calcium transient (F/F0, fold increase) that showed only a slight decrease (−9%, Figure 1C).

In line with the compromised calcium dynamics, the contractile efficiency of untreated D cells was significantly reduced, as documented by the decrease in the maximal rate of shortening (−dL/dt_max_, −25%; Figure 1G) and re-lengthening (+dL/dt_max_, −22%; Figure 1H), leading to a global prolongation of re-lengthening times, measured at 50% and 90% of re-lengthening (RL50% and RL90%, Figure 1I,J). Conversely, the fraction of shortening exhibited only a moderate decline (FS, Figure 1F). SAHA exposure was able to induce a partial or complete recovery of most functional parameters that attained values comparable to those measured in C cells (Figure 1D–J).

### 2.2. Molecular Assays

#### 2.2.1. Western Blot

**Figure 2:**
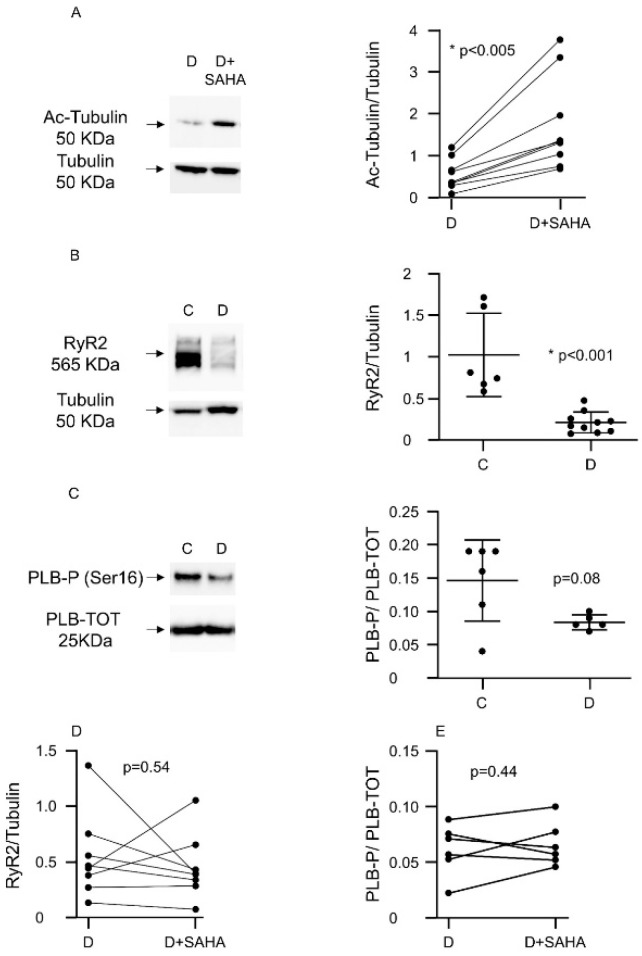
Effect of SAHA treatment on protein expression in left ventricular myocytes. (**A**) Western blot panels and densitometric analysis showing Ac-Tubulin protein expression after SAHA treatment in ventricular CMs isolated from nine D rats. Wilcoxon matched-pairs signed rank test: * *p* < 0.05 vs. C; (**B**) Western blot panels and densitometric analysis showing RyR2 protein expression reduction in CMs of diabetic rats (*n.* of rats = 10) compared to C cells (*n.* of rats = 6). Mann–Whitney U-test: * *p* < 0.001 vs. C; (**C**) Western blot panels and densitometric analysis showing the expression of phosphorylated phospholamban (PLB-P, Ser16) compared to total phospholamban (PLB-TOT), in adult C cardiomyocytes vs. D cells (*n.* of C rats = 6; *n*. of D rats = 5), normalized to total protein concentration (Ponceau). Mann–Whitney U-test: *p* = 0.08 vs. C; (**D**) Western blot panels and densitometric analysis showing RyR2 protein expression after SAHA treatment in diabetic CMs (*n*. of rats = 8). Wilcoxon matched-pairs signed rank test; (**E**) Western blot analysis showing the expression of phosphorylated phospholamban (PLB-P, Ser16) compared to total phospholamban (PLB-TOT) in adult rat CMs after SAHA treatment normalized to total protein concentration (Ponceau) (*n*. of rats = 6). Wilcoxon matched-pairs signed rank test.

We performed Western blot experiments to understand the potential molecular changes responsible for the amelioration of cardiac contractility and calcium dynamics that we observed in diabetic CMs exposed to SAHA (2.5 µmol/L). The pro-acetlylation effect of SAHA treatment was confirmed in each animal by evaluating the relative increase in acetyl-tubulin expression compared to total tubulin (Figure 2A).

In comparison with group C, untreated D cells exhibited a significant downregulation of ryanodine receptors (RyR2) (Figure 2B) and a decline of the ratio between the phosphorylated and the total form of phospholamban (PLB-P/PLB-TOT, −45%, Figure 2C), although this last difference did not reach statistical significance (*p* = 0.08). SAHA treatment did not induce the recovery of RyR2 expression levels (Figure 2D) and P-PLB/PLB-TOT ratios (Figure 2E). At this stage of the diabetic disease, the expression levels of the Sarco/Endoplasmic Reticulum Ca^2+^-ATPase (SERCA), the voltage-dependent L-type calcium channel (subunit alpha, CACNA1c) and the Na^+^/Ca^2+^ exchanger (isoform 1, NCX1) were unchanged, in both treated and untreated diabetic CMs 

#### 2.2.2. Cardiomyocyte ATP Content, Metabolic Activity, and Intracellular ROS Levels

**Figure 3:**
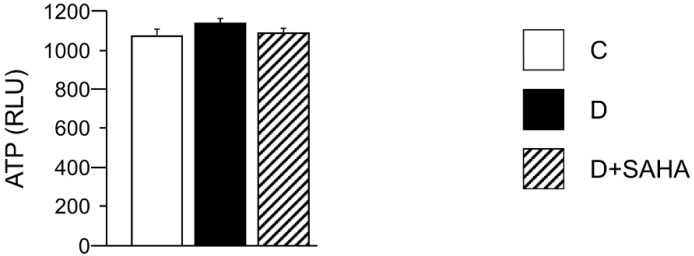
Effect of SAHA treatment on cardiomyocyte ATP content. Mean values ± SEM of ATP content in control (C), diabetic (D), and SAHA-treated D cells (D + SAHA). Values are expressed as relative light units (RLU) counts/s. Kruskal–Wallis and U Mann–Whitney test.

In order to explain, at least in part, the lower contractile efficiency and altered calcium dynamics in diabetic CMs, as well as the recovery of the cell mechanical properties induced by SAHA exposure, we measured the total intracellular ATP content. However, the ATP availability, primarily produced by the mitochondrial oxidative phosphorylation, was comparable in all groups, with only slight differences, independent of the treatment (Figure 3).

**Figure 4:**
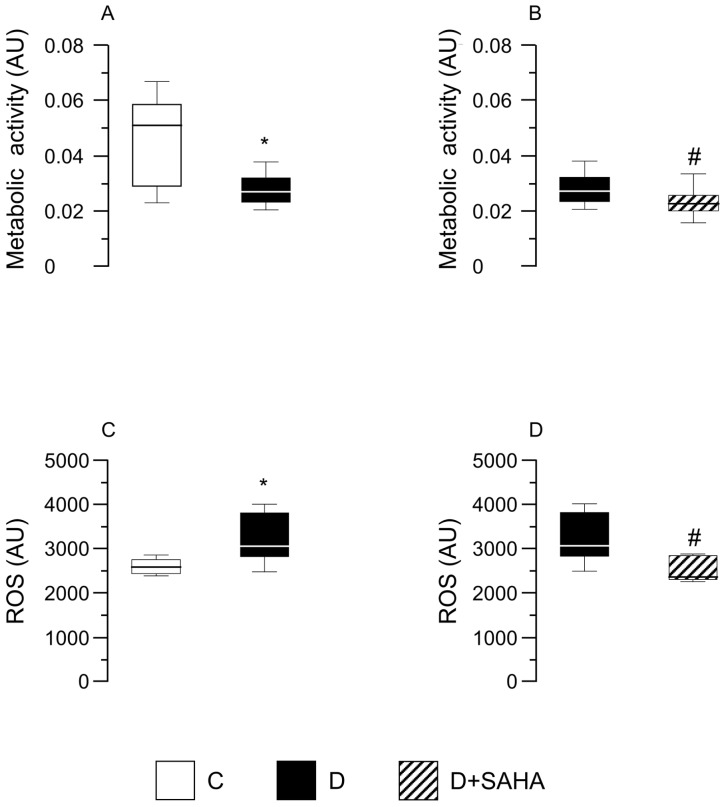
Effect of SAHA treatment on CMs’ metabolic activity and intracellular ROS content. Data are reported as median values and interquartile range. In left panels: Effect of diabetes on CM metabolic activity (**A**) and ROS levels (**C**), compared with control cells. Mann–Whitney U-test: * *p* < 0.05 vs. group C. In right panels: metabolic activity (**B**) and ROS content (**D**) measured in diabetic CMs before and after SAHA-exposure. Cells were isolated from the heart of three normal rats (group C) and five diabetic rats (groups D and D + SAHA). AU = arbitrary units. # *p* < 0.05 vs. D group; Wilcoxon matched-pairs signed rank test.

Nevertheless, when the dehydrogenase activity was analyzed, a significant (*p* < 0.05) reduction was found in D cardiomyocytes in comparison with C cells, independent of SAHA treatment (Figure 4A,B). Conversely, the exposure of D cells to SAHA (D + SAHA) completely abolished the increase in intracellular ROS content observed in untreated D cardiomyocytes, restoring the values measured in C cells (Figure 4C,D).

## 3. Discussion

Diabetes and other metabolic conditions characterized by elevated blood glucose constitute important risk factors for cardiovascular disease. Hyperglycemia per se can directly target myocardial cells and the tissue microenvironment [4], progressively leading to ineffective electro-mechanical properties of the heart at advanced stages of diabetes [21]. Hence, the importance of identifying new adjuvant pharmacological approaches able to prevent or reduce the initial cellular alterations dictating the progressive deterioration of cardiac function. In this context, the main objective of the present study was to assess whether HDAC inhibition could reverse early abnormalities occurring in CMs after a short period of hyperglycemia.

Taken together, our results suggest that at the very early stages of the diabetic disease, SAHA-induced HDAC inhibition can counteract the occurrence of the first signs of CM dysfunction mainly by reducing intracellular ROS accumulation.

We used a model of STZ-induced early diabetes widely characterized from a morpho-functional point-of-view. We confirmed that three weeks of hyperglycemia corresponds to a transitional phase preceding the overt diabetic cardiomyopathy phenotype [4,24]. At this time point, an initial deterioration of the intrinsic contractile properties of CMs associated with altered intracellular calcium dynamics occurred in D cells compared with those of group C. The slow Ca^2+^ transient decay and the parallel decrease in the rate of cell re-lengthening is indicative of either impaired sequestration of Ca^2+^ in the SR or a reduced calcium extrusion through the plasmalemma sodium/calcium exchanger (NCX), the two most important players involved in cytosolic calcium clearing [25,26]. At the molecular level, however, no substantial changes were detected in the expression levels of most proteins involved in the excitation–contraction process, namely SERCA2, NCX, and L-type calcium channels. Conversely, a significant down-regulation of RyR2 expression was observed in untreated D cells that may explain the delayed time-to-peak of the Ca^2+^ transient. This finding confirms that a decrease in RyR2 expression level, already known to occur in more advanced phases of diabetes [27], may represent an important early component of the perturbed calcium cycling of hyperglycemic myocytes [21]. The reactive oxygen species accumulation observed in D CMs can also contribute to RyR2 dysfunction by altering their redox state [28] and phosphorylation [29]. In addition, we recently showed that, at these stages of diabetes, the oxidative stress promotes a cellular inflammatory response, implying the release of Monocyte Chemoattractant protein 1 (MCP-1) and Fractalkine pro-inflammatory chemokines from parenchymal and stromal cardiac cell compartments [4]. It should be outlined that Fractalkine, besides its chemo-attractant action, can exert direct effects on the intracellular contractile machinery by binding to its receptor on the membrane of ventricular CMs [30,31]. ROS accumulation is also linked to alterations in mitochondrial function and can have noxious effects by directly acting on different redox-sensitive target molecules involved in the signal-transduction pattern, as well as proteins that participate in the excitation–contraction process, including SERCA2 whose increased oxidation results in contractile alterations [29].

Although other mechanisms cannot be excluded, these findings support the hypothesis that the positive effects induced by low-dose SAHA treatment on cardiomyocyte performance can be attributed, at least in part, to its capacity of reducing ROS accumulation, an early event in the diabetic heart following diabetes-induced metabolic changes. This interpretation is also supported by previous reports showing that scavenging of ROS improves calcium homeostasis and attenuates cardiac derangement in multiple models of heart disease, including heart failure, hypertrophy, diabetic cardiomyopathy, and aging [16,32,33]. The difference in metabolic activity between control and diabetic rats, revealed through MTS assay, could be related to an impairment of mitochondrial function induced by the oxidative stress, in particular the loss of mtDNA integrity that can have consequences on bioenergetics and metabolism [34]. A concomitant decrease in metabolic processes, mainly NAD(P)H dehydrogenase activities and ROS levels, was found in diabetic CMs after SAHA exposure, suggesting a possible mechanistic correlation among cell metabolism and altered ROS production. It could be hypothesized that SAHA helps in restoring a correct redox signaling in diabetic cells, which is essential for the maintenance of cardiomyocyte homeostasis and is involved in the cardiac response to stress [35].

In conclusion, we provided here a proof of concept that the modulation of HAT/HDAC activity can counteract early diabetes-induced oxidative stress and functional changes in CMs. Such observations constitute the starting point for further mechanistic studies aimed at identifying new adjuvant pharmacological approaches capable of preventing the initial myocardial damage occurring in the diabetic heart and its progression towards an overt, clinically relevant, pathological condition.

## 4. Materials and Methods

The investigation was approved by the Veterinary Animal Care and Use Committee of the University of Parma-Italy (31 March 2016) and conforms to the National Ethical Guidelines of the Italian Ministry of Health (Prot. N° 614/2016-PR; 17 June 2016). Euthanasia in experimental animals was performed under ketamine chloride anesthesia (Imalgene, Merial, Milan, Italy; 40 mg/kg i.p.) plus medetomidine hydrochloride (Domitor, Pfizer Italia S.r.l., Latina, Italy; 0.15 mg/kg i.p). The details of the experimental procedures reported under Section 4.2, Section 4.3, Section 4.4 and Section 4.5 have been already described [23,24,36] (see also in the Supplementary Material).

### 4.1. Animals and Experimental Study

The study population consisted of 11 male Wistar rats (Rattus norvegicus) aged 12–14 weeks, weighing 394 ± 8 g. Five animals were used as the control group (C group) while in six animals (D group), diabetes was induced by a single intra-peritoneal injection of streptozotocin (STZ, 60 mg/kg). All animals had normal blood glucose levels at the beginning of the experimental protocol (106 ± 3 mg/dL). Two days after STZ injection, glycaemia significantly rose in D rats compared with C (360 ± 50 mg/dL vs. 107 ± 4 mg/dL; *p* < 0.01). During the subsequent weeks, blood glucose levels remained stable in both C and D groups until the end of the experimental protocol (after three weeks of hyperglycemia: C = 103 ± 1 mg/dL; D: 375 ± 24 mg/dL). A 5% decrease in body weight was observed in diabetic animals during the first two weeks after STZ injection. Subsequently, body mass exhibited only negligible changes while in C animals it slightly increased (body weight at sacrifice: C = 423 ± 12 g; D: 374 ± 20 g). Three weeks after the STZ or vehicle injection, the animals were anesthetized and the heart was rapidly removed after decapitation. Ventricular CMs were enzymatically isolated for the ex-vivo measure of the functional parameters and then stored at −80 °C for subsequent molecular assay (see Section 4.4).

Twelve additional age-matched animals (7 C and 5 D) were used to increase the sample size for molecular assay and to evaluate the cardiomyocyte ATP content, metabolic activity, and ROS levels (see Section 4.4, Section 4.5, Section 4.6 and Section 4.7).

### 4.2. Myocyte Isolation

From the heart of each animal, individual ventricular myocytes were enzymatically isolated by collagenase perfusion in accordance with a procedure previously described [24]. Then, cells were used for measuring sarcomere shortening and calcium transients (IonOptix, Milton, MA, USA). Functional measurements in CMs isolated from diabetic hearts were performed before (D group) and after exposure to SAHA (2.5 µmol /L) for 90 min (D + SAHA) [23]. The concentration and the period of SAHA exposure have been selected on the basis of previous in vitro/ex-vivo studies by our group [23] and other investigators [37]. It has been shown that SAHA in the range of 1 to 5 µmol/L increases histone acetylation in an in vitro model within one hour, reaching the plateau effect at 3 h [35]. We also recently reported that 90 min are sufficient to achieve the acetylation increase of other HDAC potential targets, including cytoplasmic substrates, and to preserve cell viability [23].

### 4.3. Cardiomyocyte Mechanics and Calcium Transients

Cardiomyocyte contractile properties and calcium dynamics were simultaneously recorded from a total of 45 control, 61 D, and 48 D + SAHA isolated LV myocytes in order to measure the following parameters: the mean diastolic sarcomere length and the fraction of shortening (FS); the maximal rates of shortening and re-lengthening (±dL/dt_max_); the time at 10%, 50%, and 90% of re-lengthening (RL10%, RL50% and RL90%, respectively), the amplitude of the calcium transient (expressed as normalized fluorescence (f/f0); the time-to-peak of the calcium transients (TTP) and the time constant (tau) of the fluorescence signal decay, taken as an index of the rate of intracellular calcium clearing [25].

### 4.4. Western Blot Analysis

SDS-PAGE and immunoblot were performed as previously described [23]. The following primary antibodies were used SERCA2 (1:1000, SantaCruz Biotecnology, Dallas, TX, USA, sc-376235), Acetylated-tubulin (1:1000, Sigma-Aldrich, St. Louis, MO, USA # T7451), Tubulin (1:2000, Abcam, Cambridge, UK, # ab59680), Ryanodine Receptor (C3-33) (1:1000, Abcam # ab2827), CACNA1c (1:500, Abcam # ab81095), NCX-1 (1:250, SantaCruz Biotecnology, Dallas, TX, USA #sc-32881), Phospho-Phospholamban (Ser16) (1:5000, Merck Millipore, Burlington, MA, USA # 07-052), Phospholamban (1:8000, Abcam #ab2865). The band density was evaluated using the Image Lab software 5.2.1 (BioRad, Hercules, CA, USA) and quantifications were normalized either to total protein concentration (Ponceau red staining) or Tubulin.

### 4.5. ATP Content in Left Ventricular Myocytes

Frozen CMs isolated from two C and four D rats, either untreated or exposed to SAHA for 90 min, were resuspended in phosphate buffered solution (PBS) and aliquoted in triplicate in a 96-well white plate, as previously described [36]. The ATP intracellular content was measured by the Luminescence ATP Detection Assay System (ATPlite) (PerkinElmer, Waltham, MA, USA) according to the manufacturer’s instructions. Briefly, cells were lysed, shaken, and dark incubated with the substrate solution. Then, the luminescence intensity was measured by the EnSpire^®^ multimode plate reader (PerkinElmer) and normalized for the total protein content of each sample.

### 4.6. Cardiomyocyte Metabolic Activity

Metabolic activity was detected through CellTiter96R AQueous One Solution Cell Proliferation Assay (MTS) (Promega Corporation, Madison, WI, USA) in ventricular CMs isolated from three control and five diabetic rats. Aliquots of isolated D CMs (1.5 mL), at a final concentration of approximately 12 × 10^4^ cell/mL, were either untreated or exposed to SAHA (2.5 µmol/L) for 90 min. Afterwards, cells were re-suspended in fresh medium and 100 µL of cell suspension and were seeded, in triplicate, in a 96-well plate. Twenty microliters of the CellTiter96R AQ One Solution Cell Proliferation Assay were added to each well; cells were incubated at 37 °C in a humidified (95%), CO_2_ (5%) incubator for 4 h, and then the absorbance at 485 nm was recorded with a 96-well plate reader (TECAN SpectraFluor Plus, Männedorf, Switzerland). Differences in absorbance were correlated to different activities of the dehydrogenases.

### 4.7. ROS Assay

ROS assays were performed on aliquots of ventricular CMs isolated from the same five D rats used for detecting metabolic activity. To measure cytoplasmic ROS levels, 6 mL of CMs, at a final concentration of approximately 12 × 10^4^ cell/mL, were incubated for 30 min at 37 °C with medium containing DCFH-DA (33 µmol/L) and subsequently washed twice with PBS. SAHA-treated and untreated D cells were transferred, in triplicate, in a 96-well black plate. Fluorescence signals were recorded using a plate reader (TECAN SpectraFluor Plus, Männedorf, Switzerland) (excitation 485 nm; emission 535 nm). A positive control was performed treating cells with 0.003% H_2_O_2_ for 20 min.

### 4.8. Statistics

Normality of the data was evaluated by the D’Agostino and Pearson normality test. The results are reported as means ± standard error (SEM) or median and interquartile range for variables that were not normally distributed. The statistical treatment of data includes: General Linear Model (GLM) ANOVA for repeated measurements, Kruskal–Wallis, and U Mann–Whitney non-parametric statistical tests. For two groups, significance was assessed by Mann–Whitney U-test or Wilcoxon matched-pairs signed rank test, when appropriate (IBM-SPSS 24.0, SPSS Inc., Chicago, IL, USA). The specific test used is reported in the figure legend of each experiment. A *p*-value < 0.05 was considered statistically significant.

## Supplementary Materials

Supplementary materials can be found at https://www.mdpi.com/1422-0067/20/8/1873/s1.
