# Supplementary material for: The Histone Deacetylase Inhibitor Suberoylanilide Hydroxamic Acid (SAHA) Restores Cardiomyocyte Contractility in a Rat Model of Early Diabetes
Source: Int J Mol Sci. 2019 Apr 16;20(8):1873. doi: 10.3390/ijms20081873 (PMC6514644; doi:10.3390/ijms20081873)
Supplement: Supplementary file 1 [file ijms-20-01873-s001.pdf]

The Histone Deacetylase Inhibitor Suberoylanilide Hydroxamic Acid (SAHA) Restores Cardiomyocyte Contractility in a Rat Model of Early Diabetes

Leonardo Bocchi [1,†], Benedetta M. Motta [2,†], Monia Savi [1,†], Rocchina Vilella [1], Viviana Meraviglia [2], Federica Rizzi [3], Serena Galati [1], Annamaria Buschini [1], Mirca Lazzaretti [1], Peter P. Pramstaller [2], Alessandra Rossini [2,*] and Donatella Stilli [1,*]

Supplementary Materials and Methods
(*subheadings 4.2-4.5*)

4.2. Myocyte Isolation

From the heart of each animal, individual ventricular myocytes were enzymatically isolated by collagenase perfusion in accordance with a procedure previously described [24]. Briefly, the rat heart was removed and rapidly perfused at 37 °C by means of an aortic cannula with the following sequence of solutions: 1) a calcium-free solution for 5 min to remove the blood, 2) a low-calcium solution (0.1 mmol) plus 1 mg/mL type 2 collagenase (Worthington Biochemical Corporation, Lakewood, NJ, USA), and 0.1 mg/mL type XIV protease (Sigma, Milan, Italy) for about 20 min, and 3) an enzyme-free, low-calcium solution for 5 min. Calcium-free solution contained the following (in mmol): 126 NaCl, 22 dextrose, 5.0 $MgCl_2$, 4.4 KCl, 20 taurine, 5 creatine, 5 Na pyruvate, 1 NaH_2PO_4, and 24 HEPES (pH=7.4, adjusted with NaOH), and the solution was gassed with 100% O_2. After digestion, atria and right ventricle were dissected and the left ventricle (LV) was cut in small pieces, these fragments were filtered using a nylon mesh and re-suspended in low-calcium solution for 30 min. Then, cells were used for measuring sarcomere shortening and calcium transients. Only rod-shaped myocytes exhibiting cross striations and no spontaneous contractions were selected for physiological studies; cells were used within 8 h following enzymatic digestion. Cardiomyocytes isolated from group D hearts were either untreated or incubated with 2.5 µmol SAHA (D+SAHA) for 90 min [23] and then used for recording cell mechanics and calcium transients (IonOptix, Milton, MA, USA). A fraction of cells from each experimental group was washed three times with low-calcium solution and centrifuged (42×g for 5 min). After removing the supernatant, the pellet was stored at −80 °C for subsequent molecular assays.

4.3. Cardiomyocyte Mechanics and Calcium Transients

LV cardiomyocytes were placed in a chamber mounted on the stage of an inverted microscope (Nikon-Eclipse TE2000-U, Nikon Instruments, Florence, Italy) and superfused (1 mL/min at 37 °C) with a Tyrode solution containing (in mmol): 140 NaCl, 5.4 KCl, 1 $MgCl_2$, 5 HEPES, 5.5 glucose, and 1 $CaCl_2$ (pH 7.4, adjusted with NaOH). The cells were field stimulated at a frequency of 0.5 Hz by constant current pulses (2 ms in duration, and twice the diastolic threshold in intensity) delivered by platinum electrodes placed on opposite sides of the chamber, connected to a MyoPacer Field Stimulator (IonOptix). The stimulated myocytes were displayed on a computer monitor using an IonOptix MyoCam camera. Load-free contraction of myocytes was measured with the IonOptix system, which captures sarcomere length dynamics via a Fast Fourier Transform algorithm. Sampling rate was fixed at 1 KHz.

A total of 45 control, 61 D, and 48 D+SAHA isolated LV myocytes were analyzed to compute the following parameters: mean diastolic sarcomere length and fraction of shortening (FS), maximal rates of shortening and re-lengthening ($\pm dl/dt_{max}$), and time at 10%, 50% and 90% of re-lengthening (RL10%, RL50% and RL90%, respectively). Steady-state contraction of myocytes was achieved before data recording by means of a 10 s conditioning stimulation.

Calcium transients were measured simultaneously with cell motion. Ca^{2+} transients were detected by epifluorescence after loading the myocytes with Fluo-3 AM (10 µmol/L; Invitrogen, Carlsbad, CA, USA) for 30 min. Excitation length was 480 nm, with emission collected at 535 nm using a 40× oil objective lens (NA: 1.3). The following parameters were measured: amplitude of the calcium transient, expressed as normalized

fluorescence (f/f0: fold increase), the time to peak of the calcium transients (TTP), and the time constant (tau) of the fluorescence signal decay, taken as index of the rate of intracellular calcium clearing [25].

4.4. Western Blot Analysis

Proteins were quantified with a BCA protein kit (Thermo Fisher Scientific, MA, USA) following the manufacturer's instructions. Then, 25 μg of protein extracts were separated by SDS-PAGE on precast gradient (4–12%) gels (Invitrogen by Thermo Fisher Scientific, Waltham, Massachusetts, USA) using a MOPS running buffer (Invitrogen) and transferred onto nitrocellulose membranes (Bio-Rad, Segrate, Milan, Italy) in Transfer buffer (Life Technologies by Thermo Fisher Scientific) supplemented with 10% (v/v) methanol (Sigma-Aldrich, Milan, Italy). After blocking in 5% non-fat dry milk in PBS containing 0.1% Tween 10 (1 hour at room temperature), membranes were incubated overnight at 4 °C with primary antibodies: SERCA2 (1:1000, SantaCruz Biotecnology, Dallas, Texas, USA, sc-376235), Acetylated-tubulin (1:1000, Sigma-Aldrich # T7451), Tubulin (1:2000, Abcam # ab59680), Ryanodine Receptor [C3-33] (1:1000, Abcam # ab2827), CACNA1c (1:500, Abcam # ab81095), NCX-1 (1:250, SantaCruz Biotecnology, sc-32881), Phospho-Phospholamban (Ser16) (1:5000, Merck Millipore # 07-052), and Phospholamban (1:8000, Abcam #ab2865). Blots were washed three times in PBS-Tween buffer and then incubated with appropriate horseradish peroxidase conjugated secondary antibody (SantaCruz) for 1 hour at room temperature. Detection was performed by an enhanced chemiluminescence system (Supersignal West Dura Extended Duration Substrate, ThermoScientific). Results were quantified by Image Lab software 5.2.1 (BioRad) and quantifications were normalized either to total protein concentration (Ponceau red staining) or Tubulin.

4.5. ATP Content in Left Ventricular Myocytes

The ATP intracellular content was measured by the Luminescence ATP Detection Assay System (ATPlite) (PerkinElmer, Waltham, MA, USA) in ventricular cardiomyocytes isolated from two C and four D rats, either untreated or exposed to SAHA for 90 min. Briefly, each frozen pellet was re-suspended in 1 mL of PBS, then 60 μL of this suspension were further diluted to a final volume of 400 μL. Aliquots of 100 μL of diluted cell suspension were pipetted in triplicate in a 96-well white plate and lysed using 50 μL/well of mammalian cell lysis solution. The plate was shaken at 700 rpm for 5 minutes before adding 50 μL/well of substrate solution and being dark incubated for 10 minutes. The luminescence intensity was measured by the EnSpire® multimode plate reader (PerkinElmer). The row luminescence data were normalized for the total protein content of each sample, determined by the DC Protein assay kit (Bio-Rad, Hercules, CA, USA).
